# MicroRNAs modulated by local mIGF-1 expression in mdx dystrophic mice

**DOI:** 10.3389/fnagi.2015.00069

**Published:** 2015-05-05

**Authors:** Laura Pelosi, Angela Coggi, Laura Forcina, Antonio Musarò

**Affiliations:** ^1^Institute Pasteur Cenci-Bolognetti, DAHFMO-Unit of Histology and Medical Embryology, IIM, Sapienza University of RomeRome, Italy; ^2^Center for Life Nano Science@Sapienza, Istituto Italiano di TecnologiaRome, Italy

**Keywords:** muscular dystrophy, miRNAs, mIGF-1, muscle homeostasis, tissue niche

## Abstract

Duchenne muscular dystrophy (DMD) is a X-linked genetic disease in which the absence of dystrophin leads to progressive lethal skeletal muscle degeneration. It has been demonstrated that among genes which are important for proper muscle development and function, micro-RNAs (miRNAs) play a crucial role. Moreover, altered levels of miRNAs were found in several muscular disorders, including DMD. A specific group of miRNAs, whose expression depends on dystrophin levels and whose deregulation explains several DMD pathogenetic traits, has been identified. Here, we addressed whether the anabolic activity of mIGF-1 on dystrophic muscle is associated with modulation of microRNAs expression. We demonstrated that some microRNAs are strictly linked to the dystrophin expression and are not modulated by mIGF-1 expression. In contrast, local expression of mIGF-1 promotes the modulation of other microRNAs, such as miR-206 and miR-24, along with the modulation of muscle specific genes, which are associated with maturation of regenerating fibers and with the stabilization of the differentiated muscle phenotype. These data suggest that mIGF-1, modifying the expression of some of the active players of muscle homeostasis, is able, even in absence of dystrophin expression, to activate circuitries that confer robustness to dystrophic muscle.

## Introduction

Duchenne muscular dystrophy (DMD) is an X-linked genetic disease caused by mutations in the dystrophin gene, leading to progressive lethal muscle degeneration, chronic inflammatory response, and fibrosis (Deconinck and Dan, 2007).

Recent works have shown that micro-RNAs (miRNAs), which are important regulatory elements for proper muscle development and function (Eisenberg et al., 2009; Güller and Russell, 2010; Sharma et al., 2014), might play crucial roles in the pathogenesis of several muscular disorders, including DMD (Eisenberg et al., 2007; Chen et al., 2009). MiRNAs are small, ~22 nucleotides long, non coding RNA that function as regulatory molecules, silencing their cognate target genes (Bartel, 2004).

In a previous work, we contributed to identify a specific group of miRNAs whose expression depends on dystrophin levels and whose deregulation explains several DMD pathogenic traits (Cacchiarelli et al., 2010). This class of miRNAs, poorly expressed in mdx, was upregulated in exon-skipping-treated animals and included muscle specific (miR-1 and miR-133) and more ubiquitous (miR-29 and miR-30) miRNAs. Moreover, the negative modulation of the inflammatory miR-223 and the up-regulation of miR-29, which controls collagen deposition, was consistent with the observed amelioration of the dystrophin phenotype due to the rescue of dystrophin expression by exon-skipping approach (Cacchiarelli et al., 2010). In contrast, the restricted expression of the myomiR 206 in activated satellite cells before the onset of dystrophin synthesis, suggested that miR-206 is independent from the Dystrophin/nNOS-mediated pathway. Moreover, it has been demonstrated a common micro-RNA signature in skeletal muscle damage and regeneration induced by DMD and acute ischemia (Greco et al., 2009), suggesting an important role of miRNAs in physiopathological pathways regulating muscle response to damage and regeneration.

Among growth factors, the insulin-like growth factors 1 (IGF-1) has been implicated in many anabolic pathways in skeletal muscle. Different studies on the roles of IGF-1 isoforms in skeletal muscle growth and differentiation have provided new insights into the function of these signaling molecules in muscle homeostasis, and in the control of skeletal muscle growth and regeneration (Scicchitano et al., 2009).

We previously demonstrated that muscle restricted mIGF-1 transgene (MLC/mIGF-1) sustained muscle hypertrophy and regeneration in young and senescent skeletal muscle (Musarò et al., 2001; Pelosi et al., 2007), enhanced the recruitment of circulating stem cells in injured muscle (Musarò et al., 2004) and counteracted muscle wasting in mdx dystrophic mice (Barton et al., 2002). In particular, we found a reduction in myonecrosis and fibrosis in the muscles of mdx/mIGF-1 mice compared with age-matched mdx animals (Barton et al., 2002; Shavlakadze et al., 2004). Likewise, co−injection of the rAAV−microdystrophin and rAAV−mIGF−1 vectors resulted in increased muscle mass and strength, reduced myofibers degeneration, and increased protection against contraction−induced injury (Abmayr et al., 2005). However, no specific genes and regulatory circuitries that could account for the observed morpho-functional benefits in mdx/mIGF-1 muscle have been characterized.

In this work we took advantage of mdx/mIGF-1 mice to define whether, independently of dystrophin expression, the modulation of the dystrophic microenvironment by mIGF-1 expression has some effect on miRNAs expression.

## Materials and Methods

### Mice

Animal model used: 4 week-old C57Bl/10 (control strain) mice, 4 week-old mdx and mdx/mIGF-1 (Barton et al., 2002) mice. Mdx female mice (Jackson Laboratories) were bred with IGF-1 transgenic male mice (mIGF-1) (Musarò et al., 2001), resulting in a group of mice homozygous for the IGF-1 gene and the X-linked mdx mutation (mdx/mIGF-1) (Barton et al., 2002). Mice were maintained according to the institutional guidelines of the animal facility of the unit of Histology and Medical Embryology. All animal experiments were approved by the ethics committee of Sapienza University of Rome-Unit of Histology and Medical Embryology and were performed in accordance with the current version of the Italian Law on the Protection of Animals.

### RNA Extraction and Real Time PCR Analysis

Total RNA was prepared from liquid nitrogen powdered diaphragms homogenized with tissue lyser (QIAGEN) in TriRiagentTM (Sigma). To synthesize single-stranded cDNA, 10 ng of total RNA were reverse transcribed using the TaqMan® MicroRNA Reverse Transcription Kit (Applied Biosystem), while double-stranded cDNA was synthesized with the QuantiTect Reverse Transcription kit (Qiagen). miRNA and mRNA analysis were performed on an ABI PRISM 7500 SDS (Applied Biosystems), using specific TaqMan assays (Applied Biosystems). Relative quantification was performed using as endogenous controls U6 snRNA for miRNAs and HPRT1 for mRNAs. The relative expression was calculated using the 2-DDCt method (Livak and Schmittgen, 2001).

### Protein Extraction, Western Blot Analysis

Diaphragm muscles from at least 3 animals/strain (wild type, mdx, and mdx/mIGF-1 mice) were homogenized in modified lysis buffer (Tris-HCl, pH 7.5/20 mM, EDTA/2 mM, EGTA/2 mM, sucrose/250 mM, DTT/5 mM, Triton-X/0.1%, PMSF/1 mM, NaF/10 mM, SOV_4_/0.2 mM, cocktail protease inhibitors/1X (Sigma)). Muscle lysates were processed as previously described (Pelosi et al., 2007). Filters were blotted with antibodies against: HDAC2 (Santa Cruz Biotechnology, INC), HADAC4 (Santa Cruz Biotechnology, INC) and αTubulin (Sigma). Signals were captured by ChemiDoc-It® Imaging System (UVP, LLC) and densitometric analysis were performed with VisionWorks® LS Image Acquisition and Analysis Software (UVP, LLC).

### Statistical Analysis

Statistical analysis was performed with GraphPad Prism Software. All data, if not differently specified, were expressed as mean ± SEM. Difference among groups were assessed with one-way ANOVA with a Bonferroni post test or Dunn’s post Test, and between pairs with Mann-Whitney test or Student’s *t* test assuming two-tailed distributions. Each data shown in qRT-PCR was performed on at least four different samples/animals in biological duplicates. Sample size was predetermined based on the variability observed in preliminary and similar experiments. All experiments requiring animal models were subjected to randomization based on litter. *p* < 0.05 is considered statistically significant.

## Results

### mIGF-1 Modulates the Expression of miRNAs and Factors Associated with Regeneration

In this study, we aimed to identify miRNAs involved in the pathologic phenotype of young (4 weeks of age) mdx mice and to verify whether mIGF-1 expression was able to modulate the miRNAs’ signature of dystrophic muscle.

Dystrophic-signature miRNAs has been divided into three main classes: degenerative miRNAs (miR-1, miR-29c, and miR-135a), regeneration miRNAs (miR-31, miR-34c, miR-206, miR-335, miR-449, and miR-494), and inflammatory miRNAs (miR-222 and miR-223) (Greco et al., 2009).

Moreover, these groups of miRNAs play important and crucial roles in tissue proliferation, differentiation, and homeostasis. In particular miR-1 and miR-206, classified as myomiRs on the basis of their selective expression in skeletal and cardiac muscles, regulate muscle satellite cells proliferation and differentiation, by repressing Pax-7 (Chen et al., 2010). miR-29 regulates collagens and elastin and therefore controls fibrogenesis (van Rooij et al., 2008). miR-31 might have different roles; it is strongly induced in ischemia damaged myofibers, it plays a fundamental role in postnatal vascular repair (Greco et al., 2009; Wang et al., 2014), regulates both dystrophin expression (Cacchiarelli et al., 2011) and the progression of satellite cells toward differentiation (Crist et al., 2012). miR-34c is strongly induced in ischemia damaged myofibers (Greco et al., 2009) and it promotes cell cycle withdrawal and apoptosis (Corney et al., 2007). miR-221 and miR-222 have antiangiogenic properties and play important role in the regulation of vascular inflammation (Poliseno et al., 2006). Moreover, miR-221 and miR-222 might regulate skeletal muscle differentiation (Fasanaro et al., 2010). miR-335 and miR-449 are potent mediators of cell differentiation (Lizé et al., 2011; Tomé et al., 2011), whereas miR-494 has been proven critical for the myocytes’ adaptation and survival during hypoxia/ischemia (Han et al., 2011).

Based on the dystrophic-signature miRNAs (Greco et al., 2009) and on the specific properties of selected miRNAs, at first, we defined and compared the miRNAs expression profile of dystrophic diaphragm muscle derived from 4-week-old wild type and mdx mice.

**Figure 1:**
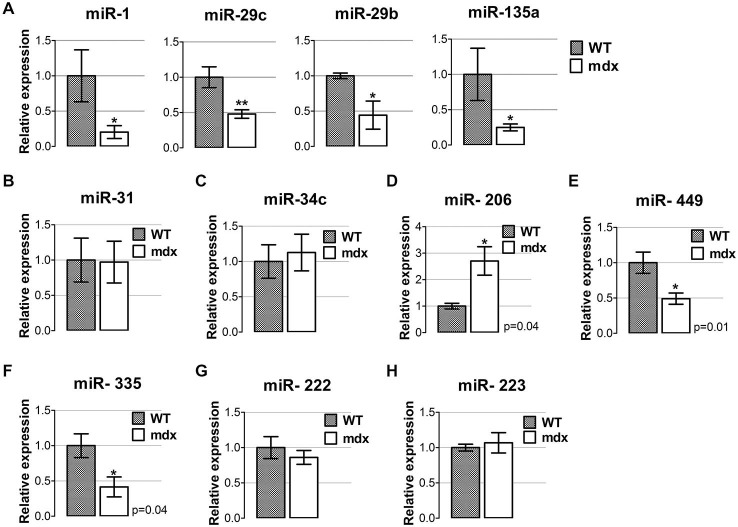
**miRNAs expression profile in diaphragm muscle of young mdx mice. (A)** qRT-PCR analysis for the expression of miRNAs classified as degenerative miRNAs (Greco et al., 2009) (miR-1, miR-29c, miR-29b and miR-135a) performed on diaphragm muscles from wild-type (WT) and mdx mice at 4 weeks of age. All miRNAs were significantly down-regulated in dystrophic diaphragm of young mdx mice compared to WT. **p < 0.005, *p < 0.05. **(B–F)** qRT-PCR analysis for the expression of miRNAs classified as regeneration miRNAs (Greco et al., 2009), such as miR-31 **(B)**, miR-34c **(C)**, miR-206 **(D)**, miR-449 **(E)** and miR-335 **(F)**, in diaphragm of 4-week old WT and mdx mice. **(G,H)** qRT-PCR analysis for the expression of miRNAs classified as inflammatory miRNAs (Greco et al., 2009), namely miR-222 **(G)** and miR-223 **(H)** in diaphragm muscles from WT and mdx mice at 4 weeks of age. For all graphs, relative expressions were normalized to U6 snRNA and shown with respect to WT set to a value of 1. Values represent mean ± SEM; *n* = 4–7 per group. *p* values using Student’s *t* test assuming two-tailed.

We observed that all degenerative- miRNAs were down-regulated in diaphragm of 4-week-old mdx compared to wild type mice (Figure 1A), whereas most of the regeneration- miRNAs reveal a differentially expression pattern in the diaphragm of 4-week-old mdx mice, compared to wild type littermates (Figures 1B–F). In particular, miR-31 and miR-34c were not modulated (Figures 1B,C); miR-206 was up-regulated (Figure 1D) and miR-449 and miR-335 were down-regulated (Figures 1E,F) in the diaphragm of 4-week-old mdx mice. The inflammatory miRNAs (miR-222 miR-223) had a similar level of expression between mdx and wild type mice. (Figures 1G,H). These data indicate that in diaphragm of young mdx mice the dystro-miRNAs have a specific pattern of expression that might correlate with the onset of dystropathology.

Muscle specific expression of mIGF-1 plays important anabolic role in skeletal muscle, promoting muscle growth and regeneration (Musarò et al., 2001, 2004; Pelosi et al., 2007) and counteracting muscle wasting in mdx dystrophic mice (Barton et al., 2002).

We verified whether mIGF-1 was able to modulate the expression of deregulated dystro-miRNAs in the diaphragm muscle of 4-week-old mdx mice.

**Figure 2:**
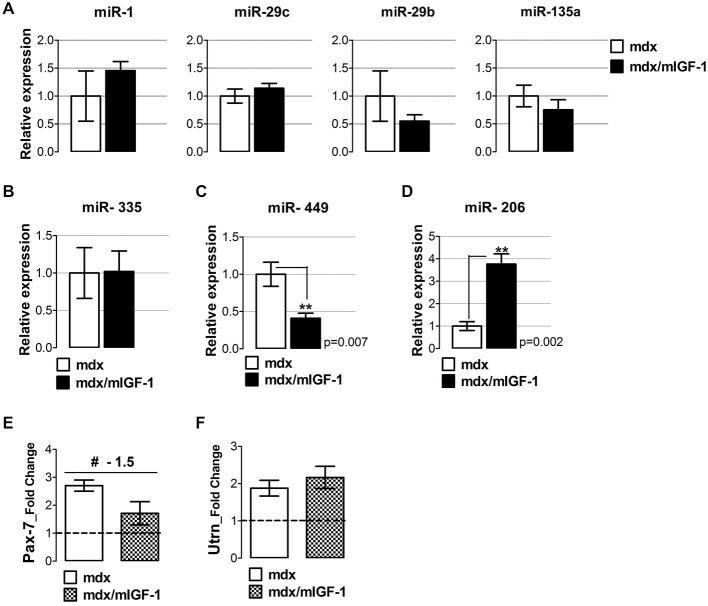
**mIGF-1 positively modulates regenerative miRNA expression in dystrophic muscle**. **(A)** Total RNA from diaphragm of 4-week-old mdx/mIGF-1 mice and mdx littermates (control), were analyzed by qRT-PCR for degenerative-miRNAs expression (miR-1, miR-29c, miR-29b and miR-135a). Relative expressions were normalized to U6 snRNA and shown with respect to mdx set to a value of 1. Values represent mean ± SEM; *n* = 5–7 per group. **(B–D)** Histograms show relative expression of regeneration- miRNAs: miR-335 **(B)**, miR-449 **(C)**, and miR-206 **(D)**, measured by qRT-PCR in diaphragm of 4-week-old mdx and mdx/mIGF-1 mice. Relative expressions were normalized to U6 snRNA and shown with respect to mdx littermates, set to a value of 1. Values represent mean ± SEM; *n* = 4–7 per group. *p* values using Student’s *t* test assuming two-tailed. **(E,F)** Graphs show the fold change of Pax-7 **(E)** and utrophin **(F)** expression levels from real time PCR analysis performed on diaphragm muscles of WT, mdx and mdx/mIGF-1 mice at 4 weeks of age. Relative expressions were normalized to HPRT1 and shown with respect to WT set to a value of 1 (dashed line). Values represent mean ± SEM; *n* = 4–6 per group. ^#^*p* < 0.05 using one way ANOVA.

QRT-PCR analysis revealed that miRNAs classified within the degenerative group, namely miR-1, miR-29b, miR-29c, and miR-135a (Greco et al., 2009) were expressed in similar manner in diaphragm muscle of both mdx and mdx/mIGF-1 mice (Figure 2A). This supports the evidence that there is a group of miRNAs that are strictly dependent on dystrophin expression (Cacchiarelli et al., 2010).

In contrast, mIGF-1 overexpression modulated regenerative miR-449 and miR-206 (Figures 2C,D) but not miR-335 expression (Figure 2B). In particular, we observed a down-regulation of miR-449 (Figure 2C), and an up-regulation of miR-206 (Figure 2D) in the diaphragm of 4-week-old mdx/mIGF-1 mice, compared to mdx littermates.

The up-regulation of the myomiR 206 suggests that mIGF-1 stabilizes the differentiated muscle phenotype, reducing the cycle of regeneration and degeneration and therefore the need to continuously activate satellite cells. We have recently demonstrated that in mdx mice, miR-206 facilitates satellite cell differentiation by restricting their proliferative potential through the repression of Pax-7 expression (Cacchiarelli et al., 2010). To support this hypothesis, we evaluated the expression of Pax-7, a marker of quiescent and activated satellite cells (Seale and Rudnicki, 2000), in both mdx and mdx/mIGF-1 mice. Pax-7 expression showed an inverse correlation with miR-206 levels, since it was lowly expressed in the diaphragm of mdx/mIGF-1 mice compared to mdx littermates (Figure 2E).

Another potential target of miR-206 is utrophin (Rosenberg et al., 2006), a gene supposed to counteract the absence of dystrophin. Real time PCR analysis revealed that utrophin was up-regulated in both mdx and mdx/mIGF-1 diaphragm (Figure 2F); however, we did not observe significant differences in utrophin gene expression between the two experimental models (Figure 2F), suggesting that the up-regulation of miR-206 did not impinge the expression of utrophin.

These data suggest that mIGF-1 might enhance the differentiation program of dystrophic fibers, inducing an up-regulation of miR-206.

### mIGF-1 Stimulates the Maturation of the Myogenic Program

**Figure 3:**
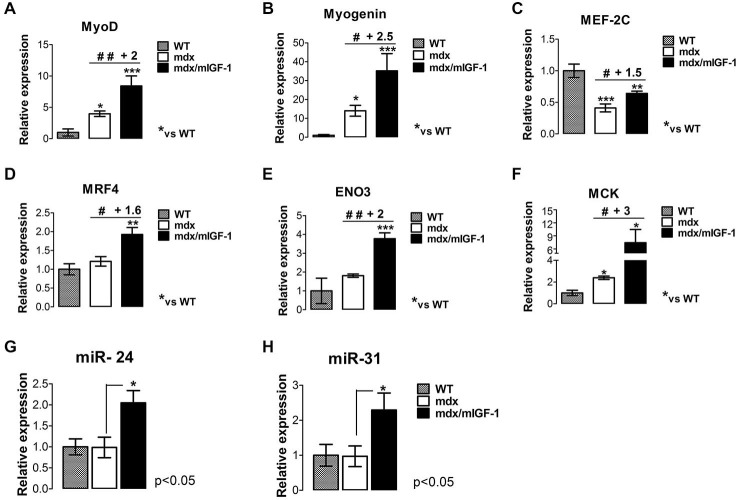
**mIGF-1 promotes maturation of the myogenic program in diaphragm muscle of mdx mice. (A–F)** Expression of MyoD **(A)**, Myogenin **(B)**, MEF-2C **(C)**, MRF4 **(D)**, β-enolase (ENO3) **(E)**, and MCK **(F)**, evaluated by real time PCR analysis in diaphragms of indicated genotypes at 4 weeks of age. Relative expressions were normalized to HPRT1 and shown with respect to WT, set to a value of 1. Values represent mean ± SEM; *n* = 4–7 per group. *p < 0.05, **p < 0.005, ***p < 0.0005, ^#^*p* < 0.05, ^##^*p* < 0.005 using one way ANOVA. **(G,H)** Histograms show miR-24 **(G)** and miR-31 **(H)** expression, analyzed by qRT-PCR in diaphragm muscles from WT, mdx, and mdx/mIGF-1 mice at 4 weeks of age. Expression levels were normalized to U6 snRNA and shown with respect to WT set to value of 1. For all graphs, values represent mean ± SEM; *n* = 5–7 per group. *p* values using one way ANOVA.

To further support this hypothesis, we evaluated other relevant markers of the myogenic program and muscle maturation. A key myogenic factor that triggers myoblast differentiation is MyoD (Tapscott, 2005; Musarò, 2014), which resulted significantly up-regulated in the diaphragm of 4-week-old mdx/mIGF-1 mice compared to that of mdx littermates (Figure 3A). Interestingly, MyoD showed a direct correlation with miR-206 levels (Figure 2D), confirming evidences that miR-206 is up-regulated by MyoD (Rosenberg et al., 2006) and targets Pax-7 mRNA (Cacchiarelli et al., 2010). Through this miR-206-mediated negative feedback mechanism, MyoD facilitates progression toward terminal differentiation (Chen et al., 2010; Hirai et al., 2010).

Myogenin is the myogenic factor that function downstream of MyoD and plays a critical role in the terminal differentiation of myoblasts (Nabeshima et al., 1993). Myogenin expression resulted significantly up-regulated in the diaphragm of mdx/mIGF-1 mice compared to mdx littermates (Figure 3B).

The final stage of skeletal muscle differentiation and maturation program is dependent on the concerted action of myogenic factors, such as MEF-2C and MRF4.

Real time PCR analysis revealed a significant increase of both MEF-2C and MRF4 transcripts in the diaphragm from 4-week-old mdx/mIGF-1 mice, compared to mdx littermates (Figures 3C,D), suggesting that mIGF-1 favors the completion of the myogenic program.

To support this hypothesis, we analyzed relevant markers of a mature muscle phenotype such as β-enolase (ENO3) and MCK, which are down-stream myogenic factors of MEF-2C and myogenin. MCK and ENO3 expression levels were significantly enhanced in the diaphragm from 4-week-old mdx/mIGF-1 mice, compared to mdx littermates (Figures 3E,F).

These data suggest that the maturation of the myogenic program, which is affected by the absence of dystrophin expression, is promoted by mIGF-1 expression.

To further support the pro-myogenic activity of mIGF-1, we analyzed the expression of another key player that functions during both differentiation and homeostatic maintenance of skeletal muscle tissues, namely the non-muscle-specific miR-24 (Sun et al., 2008). In fact, in addition to being strongly induced during myogenesis, miR-24 expression is maintained at high levels in terminally differentiated muscle tissues (Sun et al., 2008). Figure 3G shows that miR-24 was significantly up-regulated in the diaphragm of 4-week-old mdx/mIGF-1 mice compared to mdx littermates.

Of interest was also the observation that local expression of mIGF-1 enhanced miR-31 expression in the diaphragm of mdx/mIGF-1 mice, compared to mdx littermates (Figure 3H). It has been demonstrated that miR-31 targets Myf5, maintaining the quiescence of satellite cell. In activated satellite cells the levels of miR-31 are reduced, leading to Myf5 protein accumulation and satellite cells activation/proliferation (Sun et al., 2008; Crist et al., 2012).

### mIGF-1 Prevents the Activation of a Chronic Inflammatory Response

**Figure 4:**
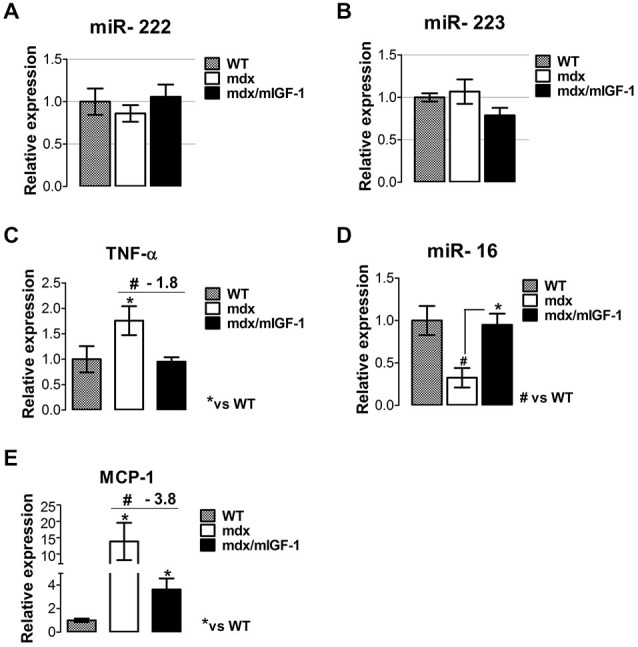
**mIGF-1 modulates factors associated with the inflammatory response in dystrophic muscle. (A,B)** Graphs show inflammatory -miR-222 **(A)** and miR-223 **(B)** expressions analyzed by qRT-PCR in diaphragm muscles from WT, mdx, and mdx/mIGF-1 mice at 4 weeks of age. Relative expressions were normalized to U6 snRNA and shown with respect to WT set to a value of 1. Values represent mean ± SEM; *n* = 5–7 per group. **(C)** Expression of TNF-α evaluated by real time PCR analysis, in diaphragms of indicated genotypes at 4 weeks of age. Relative expressions were normalized to HPRT1 and shown with respect to WT, set to a value of 1. Values represent mean ± SEM; *n* = 5–7 per group. *p < 0.05, ^#^*p* < 0.05 by one way ANOVA. **(D)** miR-16 expression measured by qRTPCR in diaphragm of 4-week-old WT, mdx and mdx/mIGF-1 mice. Relative expressions were normalized to U6 snRNA and shown with respect to WT set to a value of 1. Values represent mean ± SEM; *n* = 5–7 per group. *p < 0.05, ^#^*p* < 0.05 by one way ANOVA. **(E)** Relative expression analysis for MCP-1, measured by real time PCR in diaphragms of indicated genotypes at 4 weeks of age. Relative expressions were normalized to HPRT1 and shown with respect to WT, set to a value of 1. Values represent mean ± SEM; *n* = 5–6 per group. *p < 0.05, ^#^*p* < 0.05 by one way ANOVA.

Muscle necrosis and inflammation became significantly apparent at 3–4 weeks of age in mdx mice (Tidball and Villalta, 2010). It has been reported that miR-222 and miR-223, classified as inflammatory miRNAs (Greco et al., 2009) were highly expressed in damaged areas of the ischemic muscle and adult mdx mice, whereas they were not induced in muscles of newborn mdx mice (Greco et al., 2009). We did not observe significant change in miR-222 and miR-223 expression in mdx nor mdx/mIGF-1 mice (Figures 4A,B), suggesting that these two specific miRNAs were not modulated in the diaphragm of 4-week-old dystrophic mice and by the expression of mIGF-1.

It is known that M1 macrophages predominate during the early, acute stage of inflammation in mdx muscle (Villalta et al., 2009) and the proinflammatory cytokine tumor necrosis factor alpha (TNF-α), strongly contributes to necrosis in the dystrophin-deficient fibers of the mdx mice (De Paepe and De Bleecker, 2013). Real time PCR analysis revealed a significant down-regulation of TNF-α in the diaphragm of mdx/mIGF-1 mice, compared to mdx littermates (Figure 4C). This suggests that mIGF-1, modulating specific factors of M1 phenotype, might attenuate the severity of muscle pathology in muscular dystrophy. To further support this hypothesis we analyzed miR-16 expression, which induces TNF-α mRNA degradation (Jing et al., 2005). miR-16 was indeed strongly downregulated in the diaphragm of 4-week-old mdx compared to wild type, while its expression was rescued in mdx/mIGF-1 diaphragm (Figure 4D).

Another chemokine that mediates the cytotoxic activities of M1 macrophages in DMD is MCP-1 (Villalta et al., 2009). Real time PCR analysis revealed a significant downregulation of MCP-1 in the diaphragm of mdx/mIGF-1 mice compared to mdx littermates (Figure 4E), indicating a reduction of macrophage-dependent inflammatory response in dystrophic niche. We did not observe any modulation of the inflammatory cytokines in the diaphragm of both healthy wild type and mIGF1 transgenic mice (data not shown).

These data suggest that mIGF-1 contributes to amelioration of dystrophic niche, interfers with the activation of a chronic inflammatory program, and guarantees a functional homeostatic maintenance of dystrophic muscle.

### mIGF-1 Modulates Factors Associated with Adipogenic Differentiation

DMD is characterized by membrane fragility, myofibers necrosis and replacement of skeletal muscle by fibrous and fatty connective tissue, due to failed regeneration (Grounds et al., 2008). In order to verify whether mIGF-1 acts as an environmental cues controlling adipogenic differentiation, we analyzed relevant markers of molecular mediators of adipogenic phenotype, including PPARγ and HADCs. PPARγ is a master gene involved in adipogenic differentiation (Joe et al., 2010; Uezumi et al., 2010). In addition, it has been demonstrated that the reduction of adipogenic differentiation in young mdx mice, through Histone Deacetylase inhibition, counteracts DMD progression (Mozzetta et al., 2013; Saccone et al., 2014).

**Figure 5:**
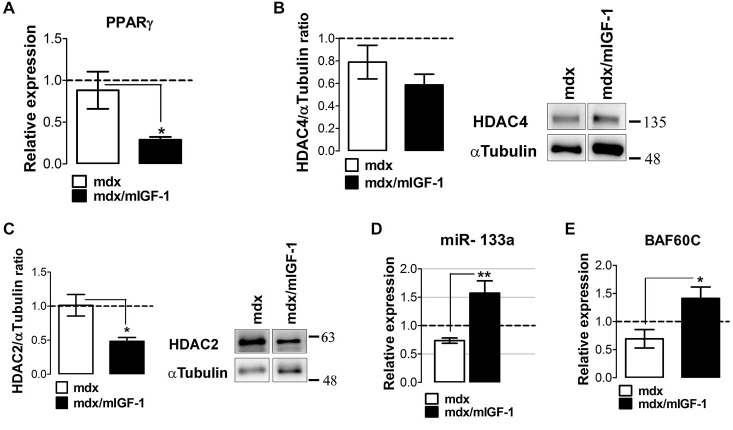
**mIGF-1 modulates factors associated with adipogenic differentiation. (A)** Graph shows the fold change of PPARγ expression levels from qPCR analysis performed on diaphragm muscles of WT, mdx and mdx/mIGF-1 mice at 4 week of age. Relative expressions were normalized to HPRT1 and shown with respect to WT, set to a value of 1 (dashed line). Values represent mean ± SEM; *n* = 4–6 per group. *p < 0.05 using one way ANOVA. **(B,C)** Representative images of western blot analysis for the expression of HADAC4 (right panel, **B**) and HADAC2 (right panel, **C**) on diaphragm of 4-week-old wild type (WT), mdx, and mdx/mIGF-1 mice. Left panels show densitometric analysis. Expression levels were normalized to HPRT1 and shown with respect to WT, set to a value of 1 (dashed line). Values represent mean ± SEM; *n* = 5–6 per group. *p < 0.05 using one way ANOVA. In **(B)** and **(C)** the lane were run on the same gel but were non contiguous. **(D)** miR-133a expression measured by qRTPCR in diaphragm of 4-week-old wild type (WT), mdx and mdx/mIGF-1 mice. Relative expressions were normalized to U6 snRNA and shown with respect to WT set to a value of 1 (dashed line). Values represent mean ± SEM; *n* = 4–7 per group. **p < 0.005, by one way ANOVA. **(E)** Graph shows the fold change of BAF60C expression levels from qPCR analysis performed on diaphragm muscles of WT, mdx and mdx/mIGF-1 mice at 4 week of age. Relative expressions were normalized to HPRT1 and shown with respect to WT, set to a value of 1 (dashed line). Values represent mean ± SEM; *n* = 4–5 per group. *p < 0.05 using one way ANOVA.

Real time PCR analysis revealed a strong down-regulation of PPARγ in the diaphragm of 4-week-old mdx/mIGF-1 mice, compared to mdx littermates (Figure 5A). Moreover, we analyzed the expression of the upstream modulators of the fibro-adipogenic progenitors (FAPs) phenotype in young mdx mice, such as HADCs and myomiRs (Colussi et al., 2008; Cacchiarelli et al., 2010; Saccone et al., 2014). While HDAC4 did not show change, we observed a significantly reduction of HADC2 in mdx/mIGF-1 compared to mdx muscle (Figures 5B,C). Moreover, myomiR-206 (Figure 2D) and myomiR-133a (Figure 5D), which ultimately lead to the activation of a pro-myogenic program at the expense of the fibro-adipogenic phenotype (Saccone et al., 2014), were strongly up-regulated in mdx/mIGF-1 compared to mdx diaphragm. The molecular mechanisms that, in concert with environmental cues, control the identity and activity of muscle cells involve the BAF60C-based SWI/SNF complex (Saccone et al., 2014). We observed a significant up-regulation of BAF60C in the muscle of mdx/mIGF-1 mice compared to mdx littermates (Figure 5E).

These data suggest that mIGF-1 creates a qualitative environment that favors muscle differentiation and maturation.

## Discussion

The major findings of this study indicate that muscle-specific expression of IGF-1 (mIGF-1) can counter aspects of the muscular dystrophy associated with the loss of dystrophin, modulating relevant molecules of the genetic and epigenetic circuitries in the mdx dystrophic mouse model.

The mdx mouse strain, lacking a functional dystrophin gene, has served as the animal model for human Duchenne and Becker muscular dystrophies (Hoffman et al., 1987). Moreover, it provides a convenient system to test possible therapeutic interventions as well as to select molecular markers that could be useful to monitor disease progression and therapeutic outcomes (Grounds et al., 2008).

The choice to analyse the effect of mIGF-1 in the diaphragm of young (4 weeks of age) dystrophic mdx mice was based on the evidence that at this age there is an acute onset of pathology (increased myofiber necrosis and elevated blood CK), in which mdx mice display muscle weakness similarly to DMD patients (Grounds et al., 2008). Moreover, the diaphragm represents one of the most severely compromised muscle in mdx mice, more closely resembling the severe pathology of DMD (Stedman et al., 1991; Lynch et al., 1997; Grounds et al., 2008).

Among epigenetic factors, miRNAs represent a class of highly conserved small molecules of about 20–23 nucleotides long that regulate gene expression at post-trasciptional level. miRNAs participate in the regulation of several essential biological processes such as cell proliferation and apoptosis, cell differentiation, stress response, and immune regulation (Sayed and Abdellatif, 2011). Several miRNAs show a tissue or developmental specific expression pattern and are present in complex regulatory networks to govern stem cells function, tissue differentiation and maintenance of cell identity during development and adult life (Fazi and Nervi, 2008). Thus, altered expression of miRNAs may be associated with different pathologies.

Several studies have analyzed the expression of miRNAs in the dystrophic mdx mouse model, (Greco et al., 2009; Cacchiarelli et al., 2010; Roberts et al., 2012). The findings point to an unexpected layer of complexity in the mdx mouse miRNA transcriptome. The differences in miRNAs expression, observed in different studies using the same mdx mouse model, can be justified considering different parameters, namely the rescue of the dystrophin-mediated signaling, the age of mdx mice, and the severity of muscle districts (McCarthy et al., 2007; Grounds et al., 2008; Yuasa et al., 2008; Greco et al., 2009; Cacchiarelli et al., 2010).

In our study, we verified whether mIGF-1 expression was able to modulate dystrophic-signature miRNAs in the diaphragm muscle of 4-week-old mdx mouse model.

It has been demonstrated that when dystrophin synthesis was restored the levels of miR-1, miR-133a, miR-29c, miR-30c, and miR-206 increased, while miR-23a expression did not change (Cacchiarelli et al., 2010). At variance with the other myomiRs, miR-206 was highly expressed in mdx as well as in exon-skypping-treated animals (Cacchiarelli et al., 2010). On the other hand, local injection of the NO-donor nitroglycerin (NTG) in mdx mice increased miR-1 and miR-29 expression, whereas did not modulate miR-206 (Cacchiarelli et al., 2010).

In our study, we revealed that mIGF-1 expression was not able to modulate miR-29 and miR-1 expression in the mdx mouse model, further indicating that the expression of these miRNAs is strictly linked to the dystrophin rescue (Cacchiarelli et al., 2010).

Our data with mdx/mIGF-1 mice also support the evidences that miR-206, which it has been demonstrated to be expressed before dystrophin synthesis starts (Cacchiarelli et al., 2010), well correlates with its expression being independent from the Dystrophin/nNOS/HDAC2 pathway and might depict the potential of muscle regeneration and maturation activated by mIGF-1.

On the contrary, McCarthy et al. (2007) proposed that increased miR-206 expression may contribute to the chronic pathology of mdx diaphragm. In addition, miR-206 seems to be primarily involved in satellite cell impairment of dystrophic dogs, although its precise role needs to be elucidated (La Rovere et al., 2014). The apparent discrepancy among these studies and our findings can be justified considering that the up-regulation of miR-206 in the diaphragm of mdx/mIGF-1 might be related to its specific function in muscle differentiation instead in muscle pathology. This consideration is supported by the evidence that the other markers of muscle differentiation and maturation are positively modulated in the mdx/mIGF-1 mice compared to mdx littermates.

The transition from cell proliferation to differentiation and maturation involves the downregulation of proliferative-associated genes. Sustained expression of Pax-7 in satellite cells delays the onset of myogenesis, and elevated expression of Pax-7 in primary myoblasts inhibits the expression of MyoD, preventing myogenin induction and muscle terminal differentiation (Olguin and Olwin, 2004; McFarlane et al., 2008).

We observed, in mdx/mIGF-1 mice compared to mdx animals, a downregulation of Pax-7 expression, associated with an increased in MyoD, myogenin, MCK and β-enolase expression, which represent relevant markers of differentiated and more mature muscle phenotype (Musarò, 2014). In addition, these data were strengthened by the up-regulation of miR-24 in mdx/mIGF-1 mice compared to mdx littermates. miR-24 is a non-muscle-specific miRNA involved in myogenesis; it is highly expressed in terminally differentiated muscle and it functions during both differentiation and homeostatic maintenance (Sun et al., 2008).

Our data are consistent with a model in which mIGF-1 stimulates muscle differentiation and maturation (Musarò and Rosenthal, 1999), by promoting the up-regulation of MyoD that, in turn, activates the expression of miR-206 (Rosenberg et al., 2006). miR-206 potently enhances the myogenic program by limiting and refining the expression of Pax-7 in myogenic progenitor cells (Chen et al., 2006, 2010; Cacchiarelli et al., 2010).

All of these data suggest that the maturation of the myogenic program and the homeostatic maintenance of dystrophic muscle tissues, which are severely affected by the absence of dystrophin expression, are facilitated by mIGF-1 expression. This might result in reduction in the cycle of regeneration and degeneration, which characterize the mdx dystrophic muscle, and therefore the need to continuously activate satellite cells. DMD is a disease of accelerated damage to muscle that causes the satellite cells to eventually be used up. Thus, mIGF-1, stabilizing the muscle phenotype, reduces the need to continuous use satellite cells, delaying the progression of disease.

Dystrophin is expressed not only in muscle cells but also in vascular endothelial cells (ECs). In DMD, the signaling defects produce inadequate tissue perfusion caused by functional ischemia due to a diminished ability to respond to shear stress induced endothelium-dependent dilation. It has been recently demonstrated that vascular densities is decreased and angiogenesis impaired in the muscles of mdx mice (Matsakas et al., 2013; Palladino et al., 2013; Shimizu-Motohashi and Asakura, 2014). Thus, increasing the density of the underlying vascular network in dystrophic muscle might be relevant therapeutic approach to reduce functional ischemia and to strength the muscle niche (Ennen et al., 2013).

In our study, we observed that mIGF-1 modulates miR-31, which is involved in vascular remodeling, regulating the activity of endothelial progenitor cells, and it plays a fundamental role in postnatal vascular repair (Greco et al., 2009; Wang et al., 2014). We can speculate that mIGF-1, modulating relevant players of vascular remodeling, might enhance vascularization in mdx mice.

Control of the inflammatory response is a critical component of efficient muscle regeneration. A balance must therefore be struck between excessive and insufficient inflammatory action. Sustained inflammatory response represents one of the pathogenic events associated with muscular dystrophy (Tidball and Wehling-Henricks, 2005; Tidball and Villalta, 2010). Release of cytokines, especially TNF-α during the inflammatory response has a strong influence on the normal progression of the proliferative stage of inflammatory cells, and on the transition from acute to chronic inflammatory response (Waheed et al., 2005; De Paepe and De Bleecker, 2013).

In our study, we revealed that mIGF-1 up-regulated miR-16, which in turn stimulates the degradation of TNF-α and the inhibition of MCP-1 expression.

Of note, TNF-α and MCP-1 are significantly associated with clinical outcome of DMD patients (De Pasquale et al., 2012).

Overall our study provides additional insights into the complex effects of mIGF-1 on muscle homeostasis and diseases and reveals the potential miRNA signature associated with mIGF-1 expression in mdx dystrophic mice. However, although these results point towards some mechanisms of action of mIGF-1, the elevated number of potential targets of these miRNAs make these mechanisms only mere suggestions.

**Figure 6:**
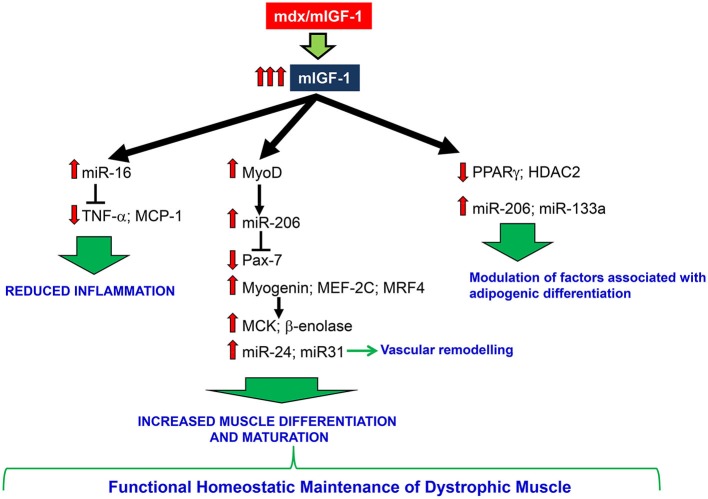
**A schematic model depicting the effects of mIGF-1 overexpression on dystrophic muscle**. mIGF-1 overexpression ameliorates dystrophic niche, reducing inflammation, modulating factors associated with adipogenic differentiation, and improving differentiation and maturation of regenerating myofibers. All of this contributes to functional homeostatic maintenance of dystrophic muscle.

Our work is consistent with a model (Figure 6) in which overexpression of mIGF-1 confers robustness to dystrophic muscle, impedes the activation of a chronic inflammatory response, activates the circuitry of muscle differentiation and maturation. This results in a functional homeostatic maintenance of dystrophic muscle.

## Conflict of Interest Statement

The authors declare that the research was conducted in the absence of any commercial or financial relationships that could be construed as a potential conflict of interest.
